# An unexpected case of a large metallic intraorbital foreign body

**DOI:** 10.5935/0004-2749.2021-0263

**Published:** 2024-06-20

**Authors:** Bangtao Yao, Gang Liu, Bei Wang

**Affiliations:** 1 Department of Ophthalmology, Nanjing Lishui People’s Hospital, Zhongda Hospital Lishui branch, Southeast University, Nanjing 211200, Jiangsu Province, China

**Figure 1:**
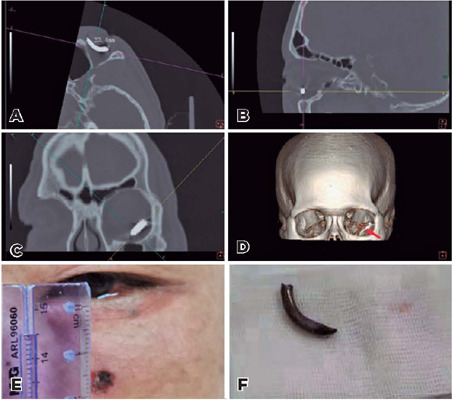
Axial (A), sagittal (B), and coronal computed tomography (CT) images (C) showing a 23.4- × 6.0-mm hyperdense plaque predominantly located in the orbit. The three-dimensional CT image demonstrated a metallic intraorbital foreign body (IOFB) despite the artifact (D, red arrow). The wound was located 20.0 mm from the lower eyelid margin (E). The IOFB (a steel nail) was removed surgically (F).

A 49-year-old man presented with conjunctival congestion without pain in the left eye in addition to ipsilateral headache. He was injured by a drill 3 days before and left the injury untreated. On his first visit to our clinic, his bestcorrected visual acuity was 6/6 in both eyes, his eyelids showed bruising, and his left eye showed subconjunctival hemorrhage and inferior-temporal conjunctival edema without lacerations. The cornea, pupils, and fundus were unremarkable. Computed tomography (CT) was performed to exclude cerebral hemorrhage and, surprisingly, revealed a hyperdense plaque in the orbit (Figure 1A B C). Three-dimensional CT demonstrated a metallic intraorbital foreign body (IOFB; Figure 1D, red arrow). After a detailed traumatic history taking, the patient suddenly complained of a healed wound on the maxillofacial region, which was covered by a mask during the coronavirus disease pandemic (20.0 mm from the eyelid margin; Figure 1E). The IOFB was removed surgically (Figure 1F). The presence of large metallic IOFBs without ocular lacerations is rare. In this case, the eyelid, cornea, and conjunctiva were intact. The high-speed IOFB entered from the maxillofacial skin into the orbit, avoiding the maxilla, orbital bone, and eyeball.

